# *Tmc2* expression partially restores auditory function in a mouse model of DFNB7/B11 deafness caused by loss of *Tmc1* function

**DOI:** 10.1038/s41598-018-29709-8

**Published:** 2018-08-14

**Authors:** Hiroshi Nakanishi, Kiyoto Kurima, Bifeng Pan, Philine Wangemann, Tracy S. Fitzgerald, Gwenaëlle S. Géléoc, Jeffrey R. Holt, Andrew J. Griffith

**Affiliations:** 1 Otolaryngology Branch, National Institute on Deafness and Other Communication Disorders (NIDCD), NIH, Bethesda, Maryland 20892 USA; 2 Departments of Otolaryngology and Neurology, F. M. Kirby Neurobiology Center, Boston Children’s Hospital, Harvard Medical School, Boston, Massachusetts, 02115 USA; 3 Anatomy and Physiology Department, Kansas State University, Manhattan, Kansas 66506 USA; 4 Mouse Auditory Testing Core Facility, NIDCD, NIH, Bethesda, Maryland 20892 USA

## Abstract

Mouse *Tmc1* and *Tmc2* are required for sensory transduction in cochlear and vestibular hair cells. Homozygous *Tmc1*^∆/∆^ mice are deaf, *Tmc2*^∆/∆^ mice have normal hearing, and double homozygous *Tmc1*^∆/∆^; *Tmc2*^∆/∆^ mice have deafness and profound vestibular dysfunction. These phenotypes are consistent with their different spatiotemporal expression patterns. *Tmc1* expression is persistent in cochlear and vestibular hair cells, whereas *Tmc2* expression is transient in cochlear hair cells but persistent in vestibular hair cells. On the basis of these findings, we hypothesized that persistent *Tmc2* expression in mature cochlear hair cells could restore auditory function in *Tmc1*^∆/∆^ mice. To express *Tmc2* in mature cochlear hair cells, we generated a transgenic mouse line, Tg[P_*Tmc1*_::*Tmc2*], in which *Tmc2* cDNA is expressed under the control of the *Tmc1* promoter. The Tg[P_*Tmc1*_::*Tmc2*] transgene slightly but significantly restored hearing in young *Tmc1*^∆/∆^ mice, though hearing thresholds were elevated with age. The elevation of hearing thresholds was associated with deterioration of sensory transduction in inner hair cells and loss of outer hair cell function. Although sensory transduction was retained in outer hair cells, their stereocilia eventually degenerated. These results indicate distinct roles and requirements for *Tmc1* and *Tmc2* in mature cochlear hair cells.

## Introduction

The primary sensory cells of the auditory organ, known as hair cells (HCs), are arranged as one row of inner HCs and three rows of outer HCs along the cochlear sensory epithelium. Inner HCs and outer HCs have different functions. Sensory receptor potentials in inner HCs induce neurotransmitter release to 95% of the auditory afferent fibers^1,2^. Thus, inner HCs function as the primary acoustic sensors. In contrast, outer HCs have few contacts with auditory afferent fibers and the role of this input remains to be identified^3,4^. Depolarization and hyperpolarization of outer HCs induces contraction and elongation of outer HC somata^5,6^. This property, known as electromotility, contributes to amplitude and frequency selectivity of sound vibrations in a process known as cochlear amplification. Electromotility and cochlear amplification depend on prestin, a transmembrane motor protein located within the outer HC lateral membrane^7,8^. Amplification can be assessed by measuring otoacoustic-emissions emerging from the cochlea with a microphone inserted in the external auditory canal.

Inner and outer HCs thus have distinct functions and it remains unknown whether this reflects differences in the molecules that mediate sensory transduction. Sensory transduction channels are non-selective cation channels whose precise molecular composition remains uncertain^9^. The opening of sensory transduction channels induces an influx of cations, predominantly potassium and calcium ions, from endolymph, the extracellular fluid that bathes sensory hair bundles in the apical membranes of HCs^10^. Endolymph has a distinctive ionic composition with low Na^+^ and Ca^2+^ concentrations and high K^+^ concentrations. The high K^+^ concentration contributes to a positive electrochemical potential, the endocochlear potential, across the HC apical surface which increases the driving force for cation influx during sensory transduction^11^. The influx of cations results in depolarizing receptor potentials which lead to the activation of voltage-gated calcium channels, neurotransmitter release and activation of glutamatergic auditory nerve fibers whose afferent terminals contact the basolateral membrane of the inner HCs^10^.

Initiation of the HC sensory transduction cascade requires expression of transmembrane channel like-1 (*TMC1*), which was identified as the gene mutated in DFNA36 and DFNB7/B11 non-syndromic hearing loss^12^. The mouse ortholog *Tmc1* also has dominant and recessive mutant alleles that cause hearing loss in mouse strains including *Beethoven* (*Bth*), *deafness* (*dn*), and mice with targeted deletion of *Tmc1* (*Tmc1*^∆/∆^)^12–14^. In general, the mouse genes and proteins required for auditory function show high similarities of sequence and functions with their human orthologs^15^.

Studies of mouse models revealed that TMC1 and the closely related TMC2 protein are located at the tips of stereocilia and are required for sensory transduction^14,16^. While the precise function of TMC1 and TMC2 remains unclear, there is substantial evidence that TMC1 and TMC2 are components of the HC sensory transduction channel^17,18^. Double homozygous *Tmc1*^∆/∆^; *Tmc2*^∆/∆^ mice showed a complete loss of peripheral auditory and vestibular function with a complete absence of sensory transduction current in structurally intact cochlear and vestibular HCs^14^. Furthermore, sensory transduction currents could be restored by viral expression of exogenous TMC1 or TMC2 in cultured *Tmc1*^∆/∆^; *Tmc2*^∆/∆^ HCs^14^. Whereas *Tmc1*^∆/∆^ (*Tmc1*^∆/∆^; *Tmc2*^+/+^) mice are deaf with normal vestibular function, *Tmc2*^∆/∆^ (*Tmc1*^+/+^; *Tmc2*^∆/∆^) mice have normal auditory function and partial loss of vestibular function detected as a reduction in the acceleration gain of the vestibulo-ocular reflex^14^.

The difference between *Tmc1*^∆/∆^ and *Tmc2*^∆/∆^ phenotypes could result from functional differences between the TMC1 and TMC2 proteins. Alternatively, it might reflect the different spatiotemporal expression patterns of *Tmc1* and *Tmc2*. *Tmc2* expression is transient in early postnatal cochlear HCs but persists in vestibular HCs, whereas *Tmc1* expression is persistent in both mature cochlear and vestibular HCs (Supplementary Fig. 1). The persistent *Tmc2* expression in vestibular HCs might thus preserve vestibular function in mature *Tmc1*^∆/∆^ mice. We therefore hypothesized that expression of *Tmc2* in mature cochlear HCs could restore auditory function in *Tmc1*^∆/∆^ mice.

In this study, we generated a transgenic mouse line, Tg[P_*Tmc1*_::*Tmc2*], in which a *Tmc2* cDNA under transcriptional control of the *Tmc1* promoter is expressed in mature cochlear HCs. The Tg[P_*Tmc1*_::*Tmc2*] transgene partially restored cochlear HC and auditory function in *Tmc1*^∆/∆^ mice, indicating that TMC1 and TMC2 have related but distinct roles for HC development, function, and survival.

## Results

### Generation of Tg[P_*Tmc1*_::*Tmc2*] mice

**Figure 1:**
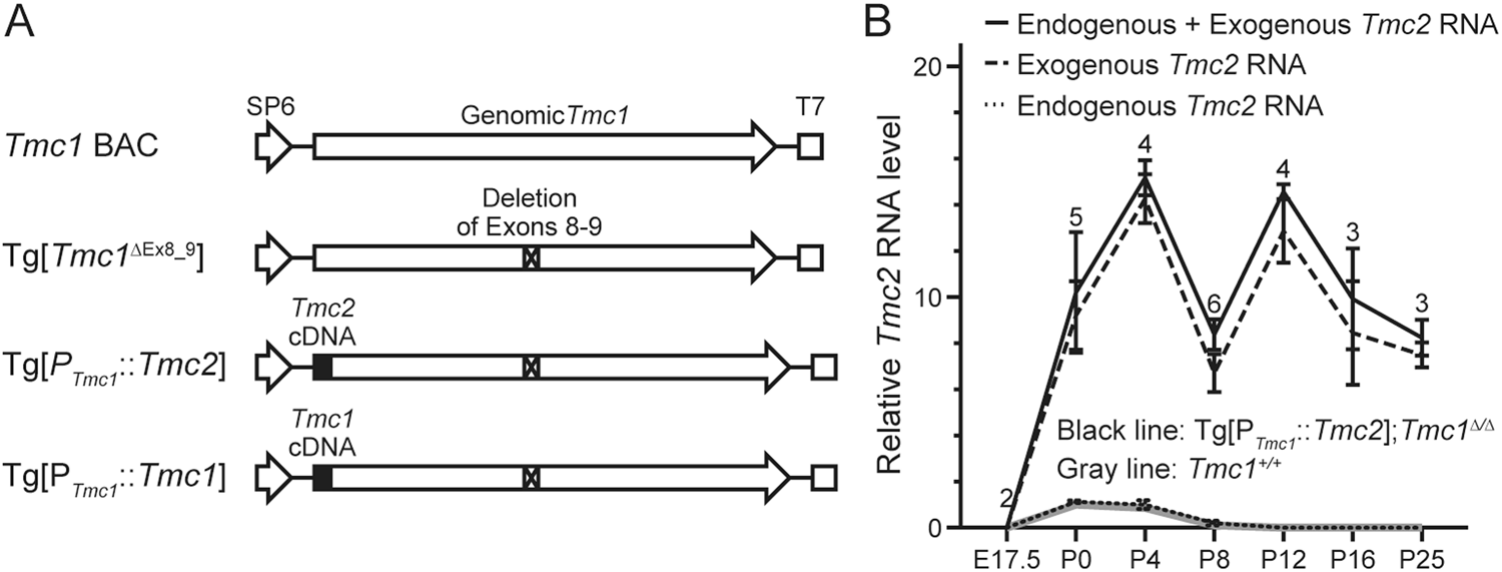
Generation of transgenic mice. (**A**) Bacterial artificial chromosome encoding wild-type mouse genomic *Tmc1* (*Tmc1* BAC) was used to construct Tg[*Tmc1*^∆Ex8_9^], Tg[P_*Tmc1*_::*Tmc2*] or Tg[P_*Tmc1*_::*Tmc1*]. To construct Tg[*Tmc1*^∆Ex8_9^], we deleted 2775 bp including exons 8 and 9. This deleted region is identical to that deleted in *Tmc1*^∆/∆^ mice^14^. To construct Tg[P_*Tmc1*_::*Tmc2*] or Tg[P_*Tmc1*_::*Tmc1*], we modified Tg[*Tmc1*^∆Ex8_9^] to replace the translation initiation codon of *Tmc1* with the *Tmc1* or *Tmc2* cDNA conjugated with the SV40 polyadenylation signal, respectively. (**B**) Relative total *Tmc2* mRNA levels (mean ± SD) of Tg[P_*Tmc1*_::*Tmc*2]; *Tmc1*^∆/∆^ cochleae (black line) and of wild-type cochleae (*Tmc1*^+/+^, gray line). In Tg[P_*Tmc1*_::*Tmc*2]; *Tmc1*^∆/∆^ cochleae, transgenic *Tmc2* mRNA (dashed line) was identified after E17.5 and remained between 8- and 16-fold levels at P25, the last time point we examined. Endogenous *Tmc2* mRNA (dotted line) was almost identical to those of wild-type cochleae. The number above each data point and bar indicates the number of mice examined. Relative RNA level was calculated by normalizing the level of each mRNA for each time point to the *Actb* level at that time point and then to the normalized *Tmc2* mRNA level in wild-type cochlea measured at P0.

In order to express TMC2 in mature cochlear HCs, we designed and generated transgenic mice, Tg[P_*Tmc1*_::*Tmc2*], in which *Tmc2* would be expressed under the control of the *Tmc1* promoter. To create the Tg[P_*Tmc1*_::*Tmc2*] transgene, we started with a bacterial artificial chromosome (BAC) clone encoding all exons of *Tmc1* and an additional 5 kb of flanking sequence both upstream and downstream of the gene. This BAC encodes the cis-regulatory elements required for inner ear expression of *Tmc1* since it restores normal auditory function in *Tmc1*^∆/∆^ mice^16^. We replaced the translation initiation codon of *Tmc1* in the BAC with a *Tmc2* cDNA and an adjacent downstream polyadenylation signal (PAS). A region including *Tmc1* exons 8–9 was deleted to prevent the expression of functional *Tmc1* from the BAC (Fig. 1A). We obtained six founder lines segregating Tg[P_*Tmc1*_::*Tmc2*] and used quantitative genomic PCR analysis to estimate the transgene copy numbers in each line. The copy numbers were 2, 2, 2, 3, 6 and 6, respectively, for lines 1, 2, 3, 4, 5 and 6. Each founder line was crossed twice with *Tmc1*^∆/∆^ mice to generate Tg[P_*Tmc1*_::*Tmc2*]; *Tmc1*^∆/∆^ mice. We performed experiments, with the exception of measurement of sensory transduction currents (measured only in line 1), in Tg[P_*Tmc1*_::*Tmc2*]; *Tmc1*^∆/∆^ lines 1, 4 and 5. In the following sections, we show the data for the Tg[P_*Tmc1*_::*Tmc2*] founder line 1, since the results were not significantly different from those with founder lines 4 or 5.

We generated other BAC transgenic mouse lines, Tg[P_*Tmc1*_::*Tmc1*] and Tg[*Tmc1*^∆Ex8_9^], as positive and negative control lines, respectively. Tg[P_*Tmc1*_::*Tmc1*] mice were used to confirm expression of *Tmc1* cDNA under the control of the *Tmc1* promoter. Tg[*Tmc1*^∆Ex8_9^] mice were used to rule out functional evidence of expression of TMC1 from Tg[P_*Tmc1*_::*Tmc2*]. We obtained three founder lines segregating Tg[P_*Tmc1*_::*Tmc1*] with estimated transgene copy numbers of 5, 6 and 17. We performed experiments with Tg[P_*Tmc1*_::*Tmc1*] lines 1 and 3 with transgene copy numbers of 5 and 17, respectively. The results for Tg[P_*Tmc1*_::*Tmc1*]; *Tmc1*^∆/∆^ line 1 are presented in the following sections since the results were not significantly different from those for line 3. We obtained six founder lines segregating Tg[*Tmc1*^∆Ex8_9^] with estimated transgene copy numbers of 2, 2, 2, 4, 10 and 14. We performed experiments with Tg[*Tmc1*^∆Ex8_9^] lines 1 and 6 with transgene copy numbers of 2 and 14, respectively. In the following sections, the results for Tg[*Tmc1*^∆Ex8_9^] line 6 are shown since the results were not significantly different from those for line 1.

### Tg[P_*Tmc1*_::*Tmc2*] expresses *Tmc2*

We examined *Tmc2* mRNA levels in Tg[P_*Tmc1*_::*Tmc2*]; *Tmc1*^∆/∆^ cochleae using TaqMan quantitative RT-PCR with target-specific probes. Transgenic and endogenous *Tmc2* mRNA were amplified separately from each other using specific primers. The reverse primer to amplify transgenic *Tmc2* mRNA hybridizes to the SV40 polyadenylation site sequence encoded by the BAC transgene, whereas the reverse primer to amplify endogenous *Tmc2* mRNA hybridizes to the genomic 3′ UTR. *Actb*-normalized data were used for relative comparison to wild-type *Tmc2* mRNA levels at P0 using the ∆∆C_t_ method. Transgenic *Tmc2* mRNA was identified after embryonic day 17.5 (E17.5) and persisted at postnatal day 25 (P25, Fig. 1B), the last time point examined. Endogenous *Tmc2* mRNA expression was not significantly different from that in wild-type *Tmc1*^+/+^ cochleae (Fig. 1B, Student’s *t*-test, *P* > 0.05). This result indicates that the *Tmc1* promoter in the BAC transgene functions to express transgenic *Tmc2* in mature cochlear HCs.

### Tg[P_*Tmc1*_::*Tmc2*] promotes cochlear hair cell survival in *Tmc1*^∆/∆^ mice

**Figure 2:**
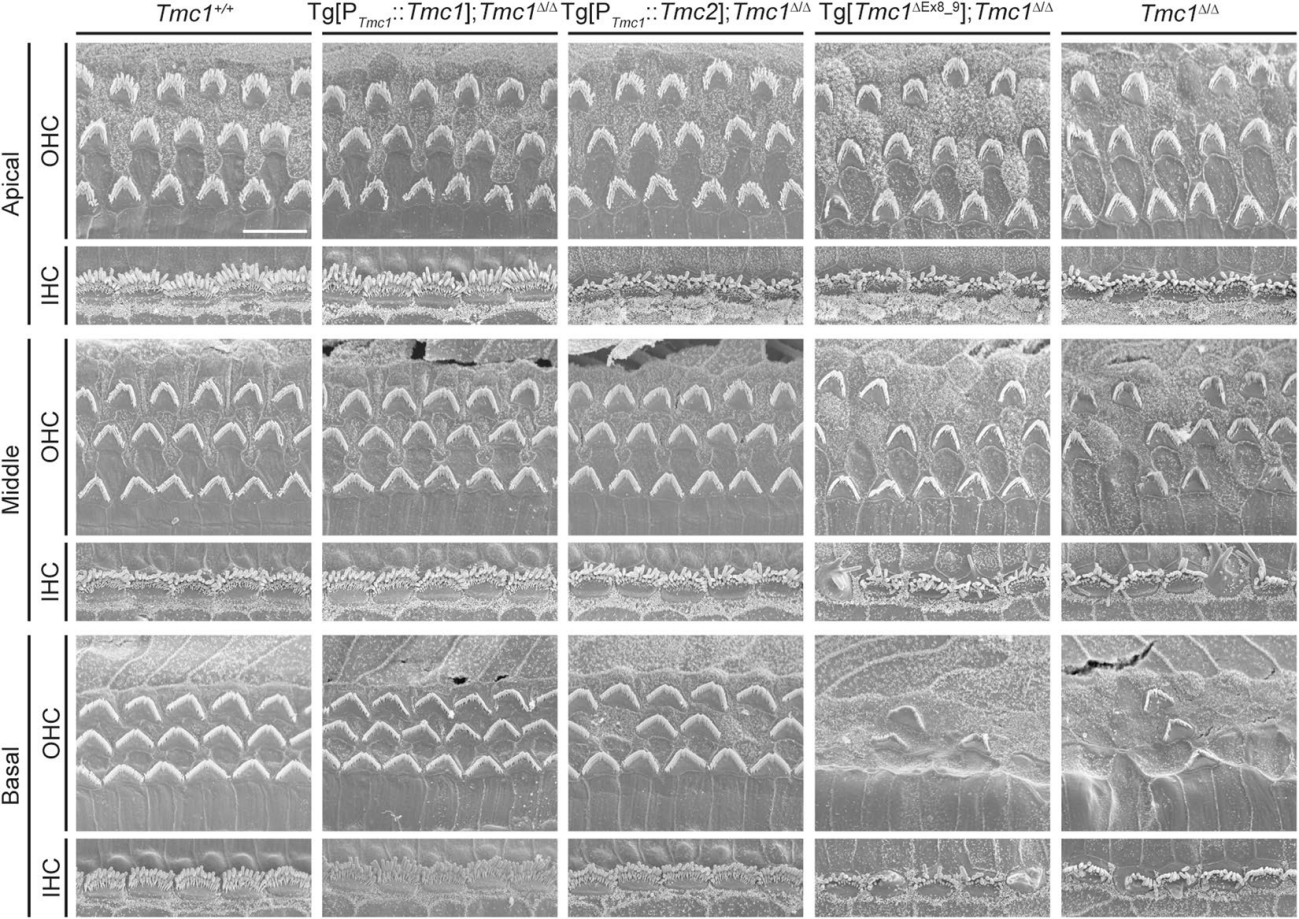
Hair cell morphology of transgenic mice examined by scanning electron microscopy. Hair cell stereocilia of wild-type (*Tmc1*^+/+^, n = 3 mice), Tg[P_*Tmc1*_::*Tmc1*]; *Tmc1*^∆/∆^ (n = 3 mice), Tg[P_*Tmc1*_::*Tmc2*]; *Tmc1*^∆/∆^ (n = 3 mice), Tg[*Tmc1*^∆Ex8_9^]; *Tmc1*^∆/∆^ (n = 4 mice), and *Tmc1*^∆/∆^ (n = 4 mice) cochleae at P25 are shown. In *Tmc1*^∆/∆^ cochleae as well as in Tg[*Tmc1*^∆Ex8_9^]; *Tmc1*^∆/∆^ cochleae, the inner HC stereocilia bundles were starting to degenerate in the middle to basal turns and the outer HC bundles were degenerating throughout the apical to basal turns. In contrast, the Tg[P_*Tmc1*_::*Tmc2*]; *Tmc1*^∆/∆^ inner HC stereocilia bundles remained intact from the apical to basal turns, whereas their outer HC stereocilia bundles were starting to degenerate. In Tg[P_*Tmc1*_::*Tmc1*]; *Tmc1*^∆/∆^ cochleae as well as wild-type cochleae, both inner and outer HC stereocilia bundles remained intact (scale bar, 10 µm).

As previously described, mutations in *Tmc1* lead to loss of normal hair bundle morphology and eventual death of both inner and outer HCs^12,13,19^. Likewise, we found that inner HC stereocilia bundles in *Tmc1*^∆/∆^ cochleae at P25 showed evidence of degeneration in middle to basal turns and that outer HC stereocilia bundles showed evidence of degeneration throughout the cochlea from apical to basal turns (Fig. 2). In contrast, Tg[P_*Tmc1*_::*Tmc2*]; *Tmc1*^∆/∆^ inner HC stereocilia bundles remained intact from the apical to basal turns (Fig. 2). Both inner and outer HC stereocilia bundles also remained intact in Tg[P_*Tmc1*_::*Tmc1*]; *Tmc1*^∆/∆^ positive control cochleae at P25 (Fig. 2). In Tg[*Tmc1*^∆Ex8_9^]; *Tmc1*^∆/∆^ negative control cochleae, inner HC stereocilia bundles showed evidence of early degeneration in the middle to basal turns and outer HC bundles were degenerating throughout the apical to basal turns, similar to *Tmc1*^∆/∆^ cochleae (Fig. 2). At earlier time points (P16), inner HC bundles remained intact, while outer HC bundles already showed evidence of degeneration in the middle to basal turns of *Tmc1*^∆/∆^ cochleae (Supplementary Fig. 2). In contrast, Tg[P_*Tmc1*_::*Tmc2*]; *Tmc1*^∆/∆^ cochleae had inner and outer HC stereocilia bundles which remained intact from apical to basal turns (Supplementary Fig. 2). These results suggest that expression of transgenic *Tmc2* promotes preservation of hair bundle morphology and survival of inner and outer HCs in Tg[P_*Tmc1*_::*Tmc2*]; *Tmc1*^∆/∆^ mice.

### Tg[P_*Tmc1*_::*Tmc2*] partially restores sensory transduction in *Tmc1*^∆/∆^ mice

**Figure 3:**
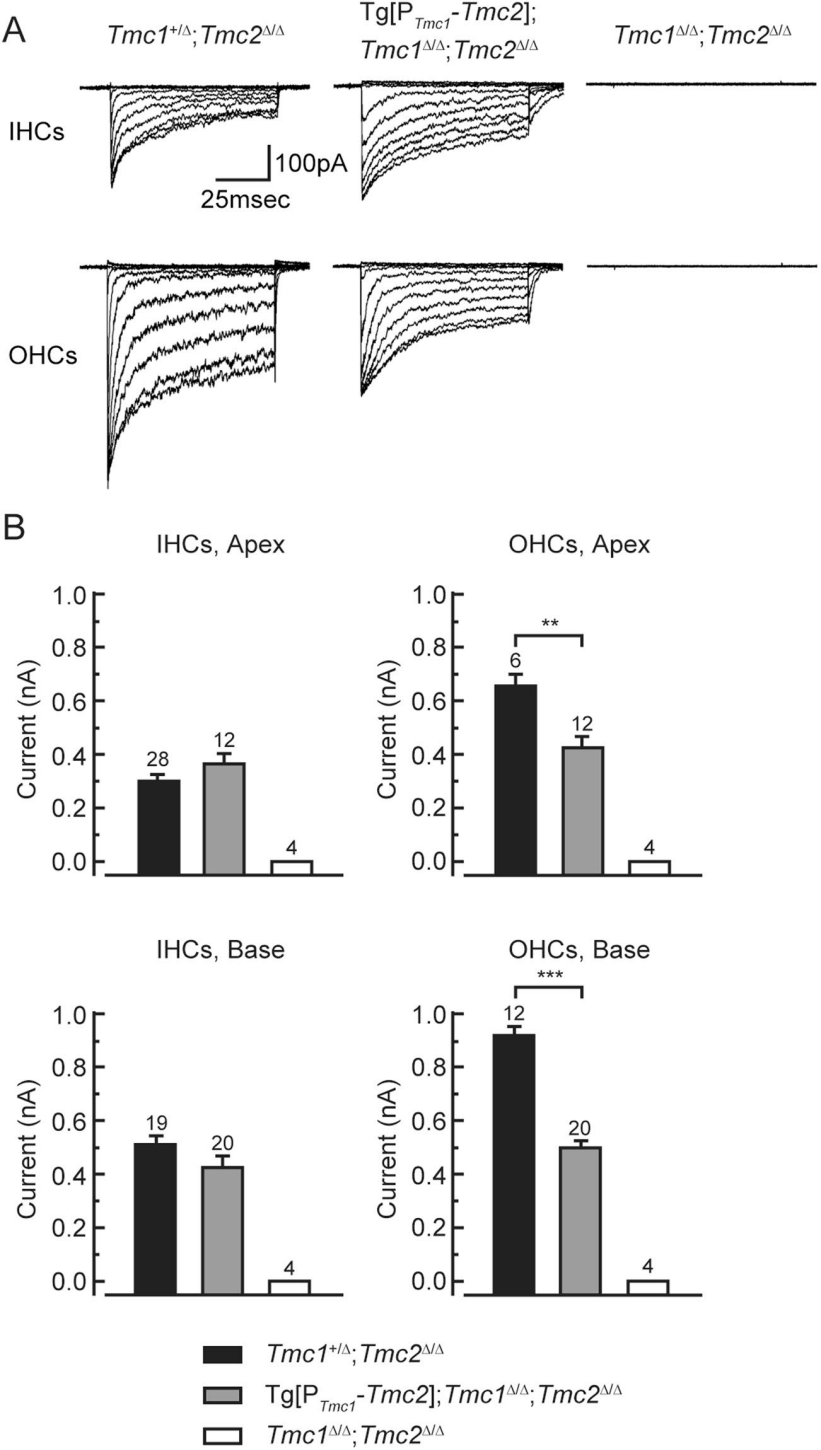
Whole-cell sensory transduction currents of Tg[P_*Tmc1*_::*Tmc2*]; *Tmc1*^∆/∆^; *Tmc2*^∆/∆^ cochlear HCs at P6-7. (**A**) Families of representative sensory transduction currents recorded from cochlear HCs of genotypes indicated above. Hair bundles were deflected between 0.4 and 1.2 microns in 0.1-micron increments from both inner HCs and outer HCs of the apical turn. The scale bar applies to all families. (**B**) The currents of Tg[P_*Tmc1*_::*Tmc2*]; *Tmc1*^∆/∆^; *Tmc2*^∆/∆^ inner HCs were not significantly different (paired *t*-test, *P* > 0.05) from those of *Tmc1*^+/∆^; *Tmc2*^∆/∆^ mice in apical and basal turns, in which only endogenous *Tmc1* is expressed. In contrast, the currents of outer HCs were significantly smaller than those of *Tmc1*^+/∆^; *Tmc2*^∆/∆^ apical (paired *t*-test, *P* < 0.01) and basal (paired *t*-test, *P* < 0.001) cochlear turns. The number above each column and bar indicates the number of HCs examined.

Since transgenic *Tmc2* expression promotes HC survival, we investigated the ability of Tg[P_*Tmc1*_::*Tmc2*] to substitute for endogenous *Tmc1* and *Tmc2* in *Tmc1*^∆/∆^; *Tmc2*^∆/∆^ cochlear HCs. We measured whole-cell sensory transduction currents in Tg[P_*Tmc1*_::*Tmc2*]; *Tmc1*^∆/∆^; *Tmc2*^∆/∆^ mice at P6-P7. In apical and basal turns, the sensory transduction currents of Tg[P_*Tmc1*_::*Tmc2*]; *Tmc1*^∆/∆^; *Tmc2*^∆/∆^ inner HCs were not significantly different from those of *Tmc1*^+/∆^; *Tmc2*^∆/∆^ inner HCs in which only endogenous *Tmc1* is expressed (Fig. 3, paired *t*-test, *P* > 0.05). In contrast, sensory transduction current amplitudes of Tg[P_*Tmc1*_::*Tmc2*]; *Tmc1*^∆/∆^; *Tmc2*^∆/∆^ outer HCs were significantly smaller than those of *Tmc1*^+/∆^; *Tmc2*^∆/∆^ outer HCs (Fig. 3, paired *t*-test, *P* < 0.01 at apical turn, *P* < 0.001 at basal turn). In vestibular type II HCs Tg[P_*Tmc1*_::*Tmc2*]; *Tmc1*^∆/∆^; *Tmc2*^∆/∆^ mice had robust sensory transduction currents that were significantly larger than those of *Tmc1*^+/∆^; *Tmc2*^∆/∆^ HCs (Supplementary Fig. 3, paired *t*-test, *P* < 0.001). These results show that transgenic expression of *Tmc2* can preserve sensory transduction currents in vestibular type II cells and cochlear inner HCs but only partially restores currents in cochlear outer HCs of postnatal *Tmc1*^∆/∆^; *Tmc2*^∆/∆^ mice during the first postnatal week.

**Figure 4:**
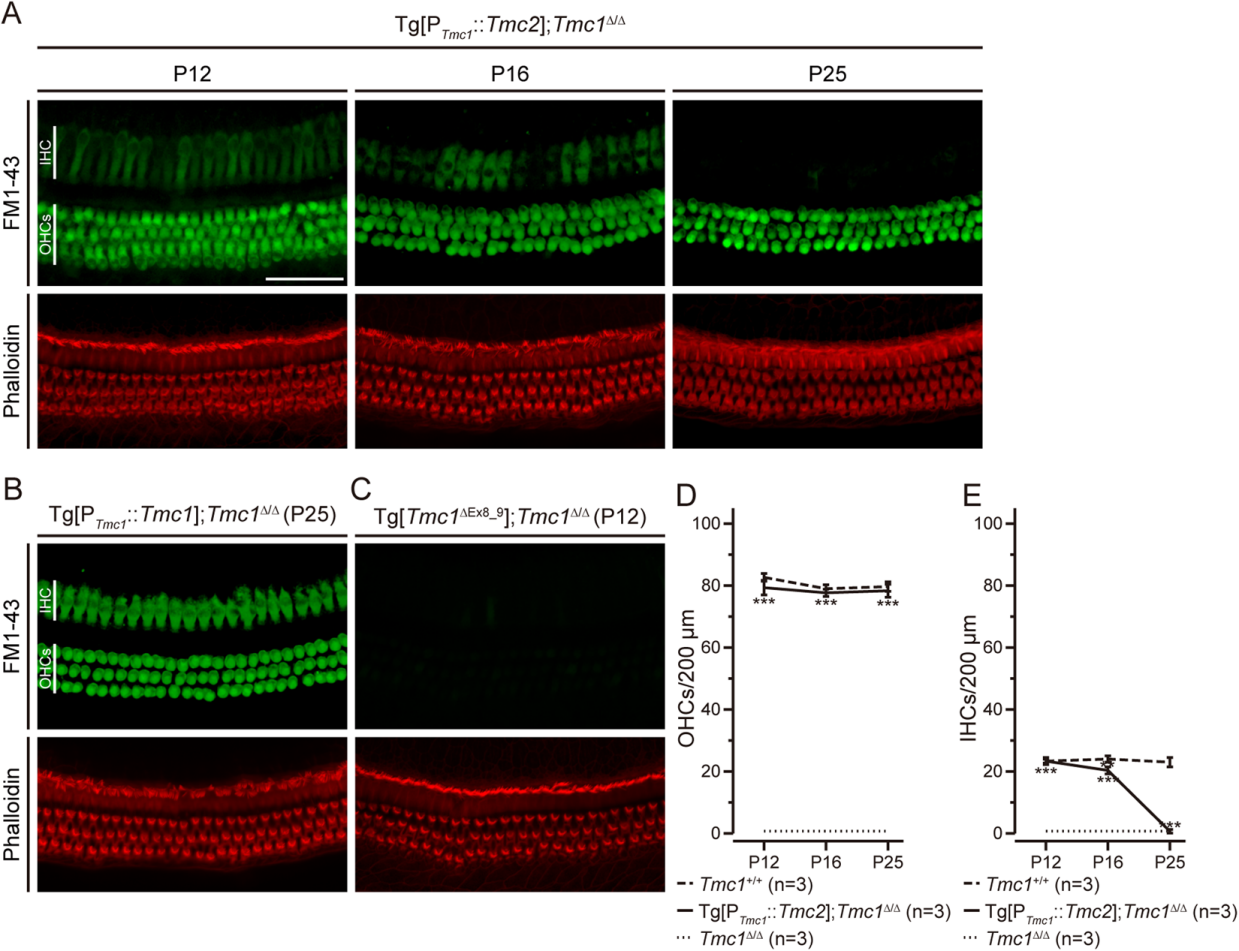
FM1-43 uptake in transgenic mouse cochlear hair cells. (**A**) FM1-43 uptake of Tg[P_*Tmc1*_::*Tmc*2]; *Tmc1*^∆/∆^ cochleae (apical turns) at P12 (n = 3 mice), P16 (n = 3 mice) and P25 (n = 3 mice). FM1-43 uptake was observed both in inner HCs and outer HCs of Tg[P_*Tmc1*_::*Tmc2*]; *Tmc1*^∆/∆^ cochleae at P12. In inner HCs, the uptake decreased with age and was limited at P25, while it remained detectable in outer HCs (Scale bar, 50 µm). (**B**) FM1-43 uptake of Tg[P_*Tmc1*_::*Tmc*1]; *Tmc1*^∆/∆^ cochleae (n = 4 mice) at P25 (apical turn). FM1-43 uptake was observed in inner and outer HCs. (**C**) FM1-43 uptake of Tg[*Tmc1*^∆Ex8_9^]; *Tmc1*^∆/∆^ cochleae (n = 3 mice) at P12 (apical turn). FM1-43 uptake was not observed in inner or outer HCs. (**D**) The mean number ( ± SD) of inner HCs in which FM1-43 uptake was observed per 200 µm length of cochlear duct is shown for Tg[P_*Tmc1*_::*Tmc2*]; *Tmc1*^∆/∆^ (black line), *Tmc1*^∆/∆^ (dotted line) or wild-type (*Tmc1*^+/+^, dashed line) cochleae at P12, P16 and P25. The number of outer HCs with FM1-43 uptake in Tg[P_*Tmc1*_::*Tmc2*]; *Tmc1*^∆/∆^ cochleae remained constant and not significantly different from those in wild-type cochleae (one-way ANOVA, *P* > 0.05) and significantly higher than the value in *Tmc1*^∆/∆^ cochleae (one-way ANOVA, *P* < 0.001) at P12, P16 and P25. (**E**) The mean number ( ± SD) of inner HCs in which FM1-43 uptake was observed per 200 µm length of cochlear duct is shown for Tg[P_*Tmc1*_::*Tmc2*]; *Tmc1*^∆/∆^ (black line), *Tmc1*^∆/∆^ (dotted line) or wild-type (*Tmc1*^+/+^, dashed line) cochleae at P12, P16 and P25. The number of inner HCs with FM1-43 uptake in Tg[P_*Tmc1*_::*Tmc2*]; *Tmc1*^∆/∆^ cochleae declined with age and disappeared at P25. It is significantly smaller than the value in wild-type cochleae at P16 (one-way ANOVA, *P* < 0.01) and is not significantly different from those of *Tmc1*^∆/∆^ cochleae (one-way ANOVA, *P* > 0.05). IHC, inner hair cells; OHCs, outer hair cells.

To examine sensory transduction at later time points, we evaluated FM1-43 uptake in HCs. FM1-43 is a fluorescent styryl dye that can enter HCs through sensory transduction channels^20,21^. Absence of FM1-43 uptake reflects loss of functional sensory transduction channels. Consistent with our previous report^14^, FM1-43 uptake was not observed in *Tmc1*^∆/∆^ cochlear HCs at P12. In contrast, FM1-43 uptake was observed in both inner and outer HCs of Tg[P_*Tmc1*_::*Tmc2*]; *Tmc1*^∆/∆^ cochleae at P12 and P16 (Fig. 4). In inner HCs, the uptake gradually decreased with age and was limited at P25, while it remained detectable in outer HCs at all ages tested (Fig. 4A; n = 3 cochleae for each of the three founder lines examined). FM1-43 uptake was observed in inner and outer HCs at P25 of Tg[P_*Tmc1*_::*Tmc1*]; *Tmc1*^∆/∆^ positive control cochleae at P25 (Fig. 4B), but not in Tg[*Tmc1*^∆Ex8_9^]; *Tmc1*^∆/∆^ negative control cochlear HCs at P12 (Fig. 4C). To quantify the FM1-43 images, we counted the number of fluorescent FM1-43-labeled HCs at P12, P16 and P25 for 200-µm sections of the cochlea and found that the numbers of FM1-43-positive outer HCs were not significantly different from those in wild-type cochleae between P12 and P25 (Fig. 4D, one-way ANOVA, *P* > 0.05), while the numbers of FM1-43-positive inner HCs declined significantly between P16 and P25 compared with those in wild-type cochleae (Fig. 4E, one-way ANOVA, *P* < 0.01 at P16, *P* < 0.001 at P25). To compare average FM1-43 intensity in inner HCs, we quantified the intensity of FM1-43 labeling in each cell and found that the average intensity in inner HCs of Tg[P_*Tmc1*_::*Tmc2*]; *Tmc1*^∆/∆^ cochleae is significantly smaller than that of Tg[P_*Tmc1*_::*Tmc1*]; *Tmc1*^∆/∆^ cochleae at P16 (Supplementary Fig. 4, one-way ANOVA, *P* < 0.005). Taken together, these data indicate that expression of transgenic *Tmc2* in mature *Tmc1*-deficient mice maintains FM1-43 uptake in outer HCs but not inner HCs, suggesting a loss of functional sensory transduction channels open at rest in inner HCs.

### Tg[P_*Tmc1*_::*Tmc2*] partially restores hearing

**Figure 5:**
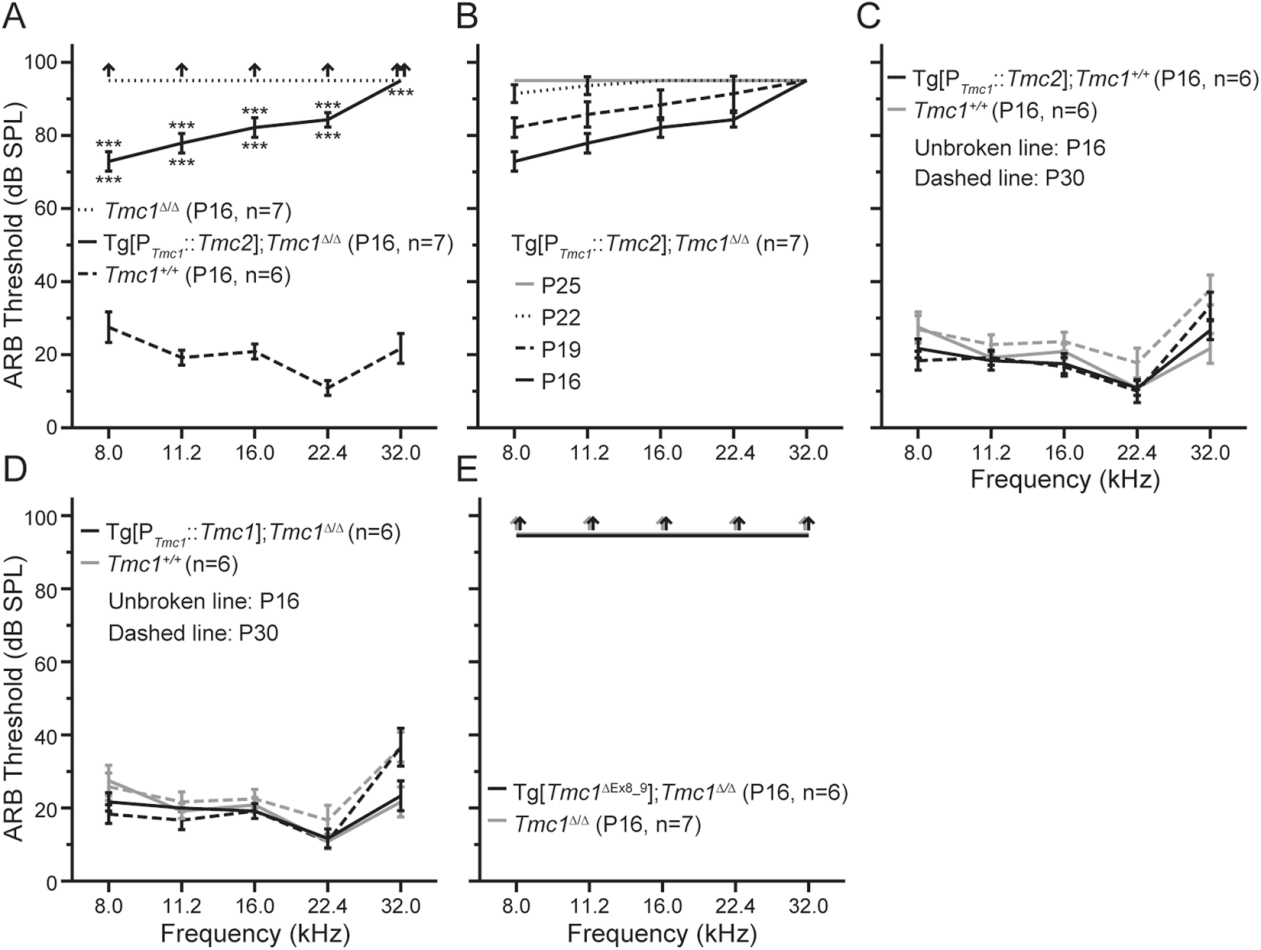
Auditory brainstem response (ABR) thresholds of better-hearing ears of transgenic mice. (**A**) ABR thresholds of Tg[P_*Tmc1*_::*Tmc*2]; *Tmc1*^∆/∆^ (mean ± SD, black line), *Tmc1*^∆/∆^ (black dotted line) and wild-type (*Tmc1*^+/+^, black dashed line) at P16. The thresholds of Tg[P_*Tmc1*_::*Tmc*2]; *Tmc1*^∆/∆^ mice were approximately 80 dB SPL, which was significantly better than those of *Tmc1*^*∆/∆*^ mice measured in response stimuli ranging from 8.0 kHz to 22.4 kHz (one-way ANOVA, *P* < 0.001). The thresholds of Tg[P_*Tmc1*_::*Tmc*2]; *Tmc1*^∆/∆^ mice were significantly higher than those of wild-type mice (one-way ANOVA, *P* < 0.001). (**B**) ABR thresholds of Tg[P_*Tmc1*_::*Tmc*2]; *Tmc1*^∆/∆^ mice at P16, P19, P22 and P25. The thresholds elevated with age until there were no detectable responses to 90 dB SPL stimuli at P25. (**C**) ABR thresholds of Tg[P_*Tmc1*_::*Tmc*2]; *Tmc1*^+/+^ and wild-type mice at P16 and P30. Their mean ABR thresholds were not significantly different from those of wild-type mice at P16 and P30 (Student’s *t*-test, *P* > 0.05). (**D**) ABR thresholds of Tg[P_*Tmc1*_::*Tmc*1]; *Tmc1*^∆/∆^ and wild-type mice at P16 and P30. The thresholds of Tg[P_*Tmc1*_::*Tmc*1]; *Tmc1*^∆/∆^ mice were not significantly different from those of wild-type mice at P16 and P30 (Student’s *t*-test, *P* > 0.05). (**E**) ABR thresholds of Tg[*Tmc1*^∆Ex8_9^]; *Tmc1*^∆/∆^ and *Tmc1*^∆/∆^ mice at P16. Responses could not be detected to 90 dB SPL stimuli at any frequencies in Tg[*Tmc1*^∆Ex8_9^]; *Tmc1*^∆/∆^ mice or *Tmc1*^∆/∆^ mice.

To investigate whether expression of transgenic *Tmc2* was capable of restoring hearing lost due to loss of endogenous *Tmc1*, we examined auditory brainstem response (ABR) thresholds in Tg[P_*Tmc1*_::*Tmc2*]; *Tmc1*^∆/∆^ mice in response to stimuli ranging from 8.0 to 32 kHz. These frequencies correspond to the apical (8.0 to 11.2 kHz), middle (16.0 to 22.4 kHz), and basal turns (32 kHz) of the cochlea. At P16, ABR thresholds were approximately 80 dB sound pressure level (SPL), which was significantly better than those of *Tmc1*^*∆/∆*^ mice (Fig. 5A, one-way ANOVA, *P* < 0.001) but significantly elevated in comparison to those of wild-type mice (one-way ANOVA, *P* < 0.001). The thresholds at all frequencies progressively increased with age until there were no detectable responses to 90 dB SPL stimuli at P25 (Fig. 5B). To investigate whether expression of transgenic *Tmc2* was detrimental for auditory function, we crossed Tg[P_*Tmc1*_::*Tmc2*] mice with wild-type mice to generate Tg[P_*Tmc1*_::*Tmc2*]; *Tmc1*^+/+^ mice. Their mean ABR thresholds were not significantly different from those of wild-type mice at P16 and P30 (Fig. 5C, Student’s *t*-test, *P* > 0.05), thus ruling out a pathogenic effect of Tg[P_*Tmc1*_::*Tmc2*] expression on wild-type HCs. Hearing thresholds of Tg[P_*Tmc1*_::*Tmc1*]; *Tmc1*^∆/∆^ (positive control) mice were not significantly different from those of wild-type mice at P16 and P30 (Fig. 5D, Student’s *t*-test, *P* > 0.05), indicating that the *Tmc1* promoter in Tg[P_*Tmc1*_::*Tmc1*] and, thus, Tg[P_*Tmc1*_::*Tmc2*] was functional. We could not detect any responses to 90 dB SPL stimuli at any frequency in Tg[*Tmc1*^∆Ex8_9^]; *Tmc1*^∆/∆^ (negative control) mice at P16 (Fig. 5E), indicating there was little or no functional expression of *Tmc1* in Tg[*Tmc1*^∆Ex8_9^]; *Tmc1*^∆/∆^ mice. Taken together, these results indicated that transgenic *Tmc2* expressed in juvenile cochlear HCs can slightly but significantly restore hearing at stimulus frequencies corresponding to the apical to middle turns of the cochlea but is unable to compensate for loss of endogenous *Tmc1* by P25.

### Tg[P_*Tmc1*_::*Tmc2*] fails to restore otoacoustic emissions in *Tmc1*^∆/∆^ mice

**Figure 6:**
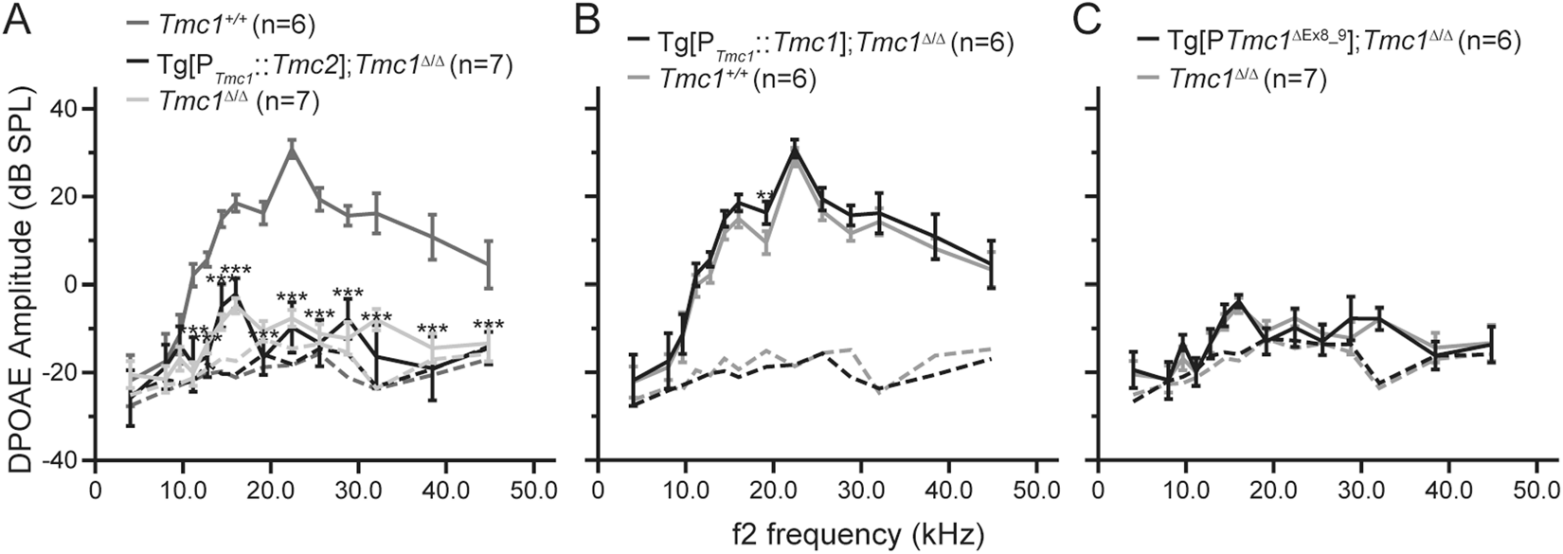
DPOAE amplitudes of transgenic mice. (**A**) Mean DPOAE amplitudes ( ± SD) of Tg[P_*Tmc1*_::*Tmc2*]; *Tmc1*^∆/∆^ (black line), *Tmc1*^∆/∆^ (light gray line) and wild-type (*Tmc1*^+/+^, dark gray line) mice at P16. The mean DPOAE amplitudes of Tg[P_*Tmc1*_::*Tmc2*]; *Tmc1*^∆/∆^ mice were significantly smaller than those of wild-type mice measured in response to f_2_ stimuli ranging from 11.2 to 44.8 kHz (one-way ANOVA, *P* < 0.001) and were not significantly different from those of *Tmc1*^*∆/∆*^ mice (one-way ANOVA, *P* > 0.05). (**B**) DPOAE amplitudes of Tg[P_*Tmc1*_::*Tmc1*]; *Tmc1*^∆/∆^ and wild-type mice at P16. The DPOAE amplitudes of Tg[P_*Tmc1*_::*Tmc1*]; *Tmc1*^∆/∆^ mice were not significantly different from those of wild-type mice except for the responses to the stimulus with f_2_ = 19.2 kHz (Student’s *t*-test, *P* > 0.05). (**C**) DPOAE amplitudes of Tg[*Tmc1*^∆Ex8_9^]; *Tmc1*^∆/∆^ and *Tmc1*^∆/∆^ mice at P16. The DPOAE amplitudes of Tg[*Tmc1*^∆Ex8_9^]; *Tmc1*^∆/∆^ mice were not significantly different from those of *Tmc1*^∆/∆^ mice (Student’s *t*-test, *P* > 0.05). Dashed lines show background noise levels in all panels.

Since transgenic *Tmc2* allowed for robust FM1-43 uptake in P16 outer HCs of Tg[P_*Tmc1*_::*Tmc2*]; *Tmc1*^∆/∆^ mice, we measured distortion-product otoacoustic emissions (DPOAEs) to assay outer HC function at the system level. Normal DPOAEs are an indication of cochlear amplification and are required for normal auditory function. Using sound stimuli ranging from 11.2 to 44.8 kHz, we found that DPOAEs were undetectable in Tg[P_*Tmc1*_::*Tmc2*]; *Tmc1*^∆/∆^ and *Tmc1*^*∆/∆*^ mice (Fig. 6A). The mean DPOAE amplitudes of Tg[P_*Tmc1*_::*Tmc2*]; *Tmc1*^∆/∆^ mice were significantly smaller than those of wild-type mice (Fig. 6A, one-way ANOVA, *P* < 0.001) and not significantly different from those of *Tmc1*^*∆/∆*^ mice (Fig. 6A, one-way ANOVA, *P* > 0.05). The mean DPOAE amplitudes of Tg[P_*Tmc1*_::*Tmc1*]; *Tmc1*^∆/∆^ mice were not significantly different from those of wild-type mice at P16 except for that measured in response to stimuli at 19.2 kHz (Fig. 6B, Student’s *t-*test, *P* > 0.05). The mean DPOAE amplitudes of Tg[*Tmc1*^∆Ex8_9^]; *Tmc1*^∆/∆^ mice were not significantly different from those of *Tmc1*^∆/∆^ mice (Fig. 6C, Student’s *t*-test, *P* > 0.05). These results indicate that expression of transgenic *Tmc2* in mature *Tmc1*-deficient cochlear outer HCs did not restore cochlear amplification. The elevated ABR thresholds of Tg[P_*Tmc1*_::*Tmc2*]; *Tmc1*^∆/∆^ mice at P16 is likely to be partially due to loss of active amplifier function of cochlear outer HCs.

### Secondary consequences of transgenic *Tmc2* expression in *Tmc1*^*∆/∆*^ inner ears

**Figure 7:**
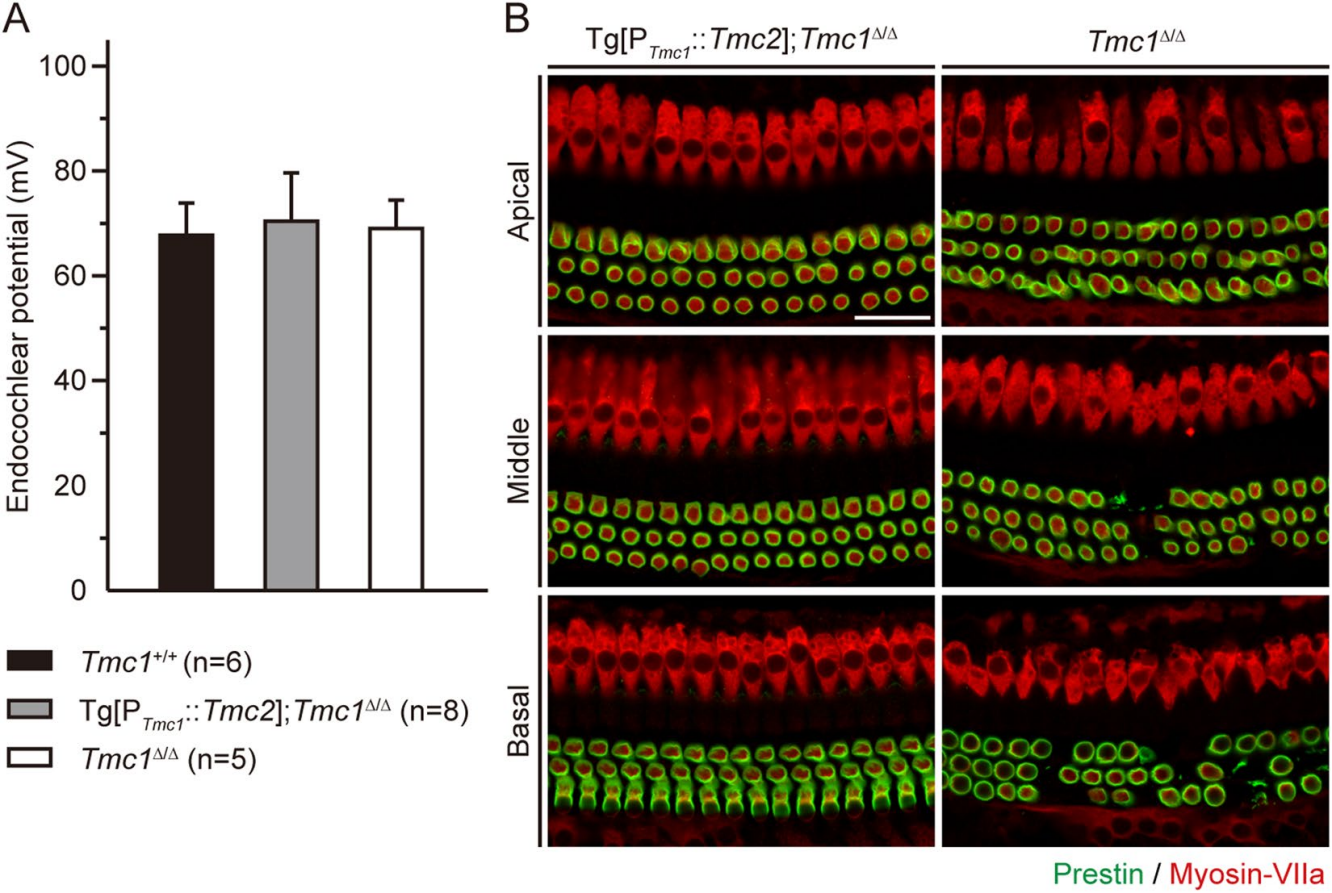
Secondary effects of Tg[P_*Tmc1*_::*Tmc2*] in *Tmc1*^∆/∆^ mice. (**A**) Normoxic endocochlear potentials (mean ± SD) of Tg[P_*Tmc1*_::*Tmc2*]; *Tmc1*^∆/∆^ (gray bar), *Tmc1*^∆/∆^ (white bar) and wild-type (*Tmc1*^+/+^, black bar) mice at P16. Normoxic endocochlear potentials of both Tg[P_*Tmc1*_::*Tmc2*]; *Tmc1*^∆/∆^ and *Tmc1*^∆/∆^ mice were not significantly different from those of wild-type mice (one-way ANOVA, *P* > 0.05). (**B**) Prestin expression in Tg[P_*Tmc1*_::*Tmc2*]; *Tmc1*^∆/∆^ (n = 4 mice) and *Tmc1*^∆/∆^ cochleae (n = 4 mice) at P16. Prestin immunoreactivity^38^ was identified along the perimeter of outer HCs from the apical to basal turns of both Tg[P_*Tmc1*_::*Tmc2*]; *Tmc1*^∆/∆^ and *Tmc1*^∆/∆^ cochleae at P16. In *Tmc1*^∆/∆^ cochleae, outer HCs were degenerating in the middle to basal turns. Inner and outer HCs were counter stained with myosin-VIIa (red; scale bars, 25 µm).

Although transgenic *Tmc2* provided partial recovery of sensory transduction, the recovery of auditory function was limited and transient. As such, we investigated several additional aspects that may be secondary consequences of disrupted *Tmc1* and *Tmc2* expression. To investigate whether a loss of the endocochlear potential contributed to the abnormal auditory function in Tg[P_*Tmc1*_::*Tmc2*]; *Tmc1*^∆/∆^ mice, we measured the endocochlear potential in normoxic conditions. The endocochlear potential of both Tg[P_*Tmc1*_::*Tmc2*]; *Tmc1*^∆/∆^ and *Tmc1*^∆/∆^ mice was ~70 mV, not significantly different from that of wild-type mice at P16 (Fig. 7A, one-way ANOVA, *P* > 0.05), indicating that loss of the auditory function of Tg[P_*Tmc1*_::*Tmc2*]; *Tmc1*^∆/∆^ cannot be attributed to changes in the endocochlear potential.

To investigate why DPOAEs were not restored in Tg[P_*Tmc1*_::*Tmc2*]; *Tmc1*^∆/∆^ mice, we evaluated expression of prestin^7,8^. Anti-prestin immunoreactivity was identified along the basolateral membranes of outer HCs from apical to basal turns of both Tg[P_*Tmc1*_::*Tmc2*]; *Tmc1*^∆/∆^ and *Tmc1*^∆/∆^ cochleae at P16 (Fig. 7B), indicating that loss of DPOAEs in Tg[P_*Tmc1*_::*Tmc2*]; *Tmc1*^∆/∆^ outer HCs cannot be attributed to loss of prestin expression.

**Figure 8:**
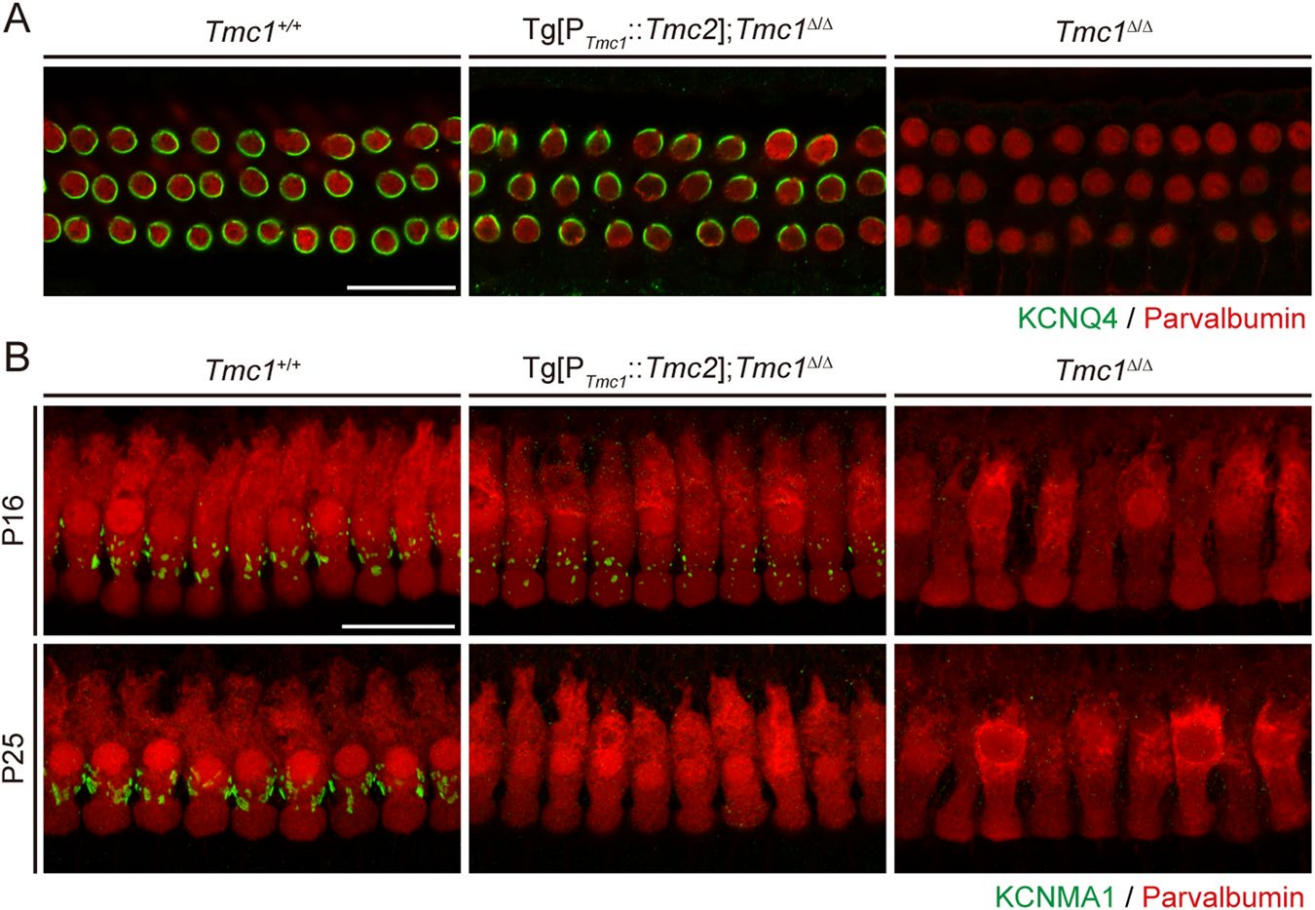
KCNQ4 and KCNMA1 expression in Tg[P_*Tmc1*_::*Tmc2*]; *Tmc1*^∆/∆^ hair cells. (**A**) KCNQ4 immunoreactivity^38^ in Tg[P_*Tmc1*_::*Tmc2*]; *Tmc1*^∆/∆^ (n = 3 mice), wild-type (*Tmc1*^+/+^, n = 3 mice), or *Tmc1*^∆/∆^ (n = 3 mice) outer HCs in the apical cochlear turn at P25 (apical turn). KCNQ4 was detected around the perimeter of outer HCs in Tg[P_*Tmc1*_::*Tmc2*]; *Tmc1*^∆/∆^ mice as well as in wild-type mice, while it was not detected in *Tmc1*^∆/∆^ mice. Hair cells were counter-stained with parvalbumin (red). (**B**) KCNMA1 immunoreactivity^38^ in Tg[P_*Tmc1*_::*Tmc2*]; *Tmc1*^∆/∆^ (n = 3 mice each at P16 and P25), wild-type (*Tmc1*^+/+^, n = 3 mice each at P16 and P25), or *Tmc1*^∆/∆^ (n = 2 mice each at P16 and P25) inner HCs in the apical cochlear turn at P16 and P25. KCNMA1 was detected as typical punctate staining at the neck of inner HCs in wild-type mice at P16 and P25. In Tg[P_*Tmc1*_::*Tmc2*]; *Tmc1*^∆/∆^ mice, KCNMA1 was detected at P16, but not at P25. In *Tmc1*^∆/∆^ mice, KCNMA1 was not detected at P16 or P25. Hair cells were counter-stained with parvalbumin (red; scale bars, 25 µm).

Prior work showed that, during postnatal maturation, outer HCs fail to acquire a slow delayed rectifier outward potassium current, *I*_k,n_, in *Tmc1* mutant (*deafness)* mice^19^. The voltage-dependent potassium channel KCNQ4 is responsible for *I*_k,n_ and is expressed in outer HCs from approximately P6 onwards^22^. Consistent with these findings, KCNQ4 immunoreactivity was undetectable in *Tmc1*^∆/∆^ outer HCs at P25 (Fig. 8A). However, we detected prominent KCNQ4 immunoreactivity in Tg[P_*Tmc1*_::*Tmc2*]; *Tmc1*^∆/∆^ outer HCs that was similar to that of wild-type outer HCs (Fig. 8A). These findings indicate that transgenic *Tmc2* preserves expression of KCNQ4 in cochlear outer HCs lacking *Tmc1*.

Finally, we asked if the auditory dysfunction in Tg[P_*Tmc1*_::*Tmc2*]; *Tmc1*^∆/∆^ mice may be due to incomplete inner HC maturation. It has previously been shown that inner HCs of *Tmc1* mutant mice fail to acquire the Ca^2+^-activated outward potassium current *I*_k,f_ carried by BK channels^19^. *I*_k,f_ is first detectable at approximately P12 in wild-type mice and is required for normal inner HC function^23,24^. We detected immunoreactivity of the pore-forming subunit of BK channels, KCNMA1, as typical punctate staining at the neck of inner HCs^25–27^ in P16 and P25 wild-type mice (Fig. 8B). KCNMA1 immunoreactivity was not detected at either age in *Tmc1*^∆/∆^ mice, consistent with the finding that inner HCs failed to acquire *I*_k,f_ in *Tmc1* mutant mice^19^. However, KCNMA1 was present in inner HCs of Tg[P_*Tmc1*_::*Tmc2*]; *Tmc1*^∆/∆^ mice at P16 but was not detected at P25, suggesting that Tg[P_*Tmc1*_::*Tmc2*]; *Tmc1*^∆/∆^ began to mature, but reverted to an immature phenotype. These results indicate that transgenic *Tmc2* can substitute for endogenous *Tmc1* for the initial acquisition, but not maintenance, of KCNMA1 expression and *I*_k_,f in inner HCs.

## Discussion

In this study, we sought to test the hypothesis that persistent *Tmc2* expression could substitute for loss of *Tmc1* expression in mature cochlear HCs in a mouse model of *Tmc1*-deficient deafness. Transgenic expression of *Tmc2* in cochlear HCs transiently and partially restored ABR thresholds in Tg[P_*Tmc1*_::*Tmc2*]; *Tmc1*^∆/∆^ mice, indicating that *Tmc2* can partially but not fully compensate for loss of *Tmc1* in mature cochlear HCs. Cochlear HC structure and function in Tg[P_*Tmc1*_::*Tmc2*]; *Tmc1*^∆/∆^ mice were also incompletely restored.

Our experiments revealed different results in inner and outer HCs. In Tg[P_*Tmc1*_::*Tmc2*]; *Tmc1*^∆/∆^ inner HCs, FM1-43 uptake gradually decreased with age from P12, and was limited at P25. This result differs from that reported by Asai *et al*.^28^, who detected small but steady sensory transduction currents in inner HCs expressing TMC2 but not TMC1 at P25. The lack of uptake of FM1-43 in our experiments was confirmed in three cochleae for each of three founder lines of Tg[P_*Tmc1*_::*Tmc2*]; *Tmc1*^∆/∆^, indicating this difference from the result in Asai *et al*.^28^ is reproducible. Since FM1-43 uptake depends on channels open at rest, it is possible that their open probability was reduced at P25 but at least some stimulus-evoked sensory transduction remained unaffected. Although FM1-43 slowly labels HCs through the conventional uptake by endocytosis^29,30^, the absence of FM1-43 uptake in Tg[*Tmc1*^∆Ex8_9^]; *Tmc1*^∆/∆^ negative control cochlear HCs indicates that the labeling of HCs by bathing cochleae with the dye was caused by FM1-43 entering through sensory transduction channels.

Immunoreactivity of KCNMA1, a pore forming subunit of the BK channel associated with *I*_k,f_ in mature inner HCs, was detected at P16, but disappeared by P25. Scanning electron microscopy revealed that the inner HC stereocilia bundles remained intact from the apical to basal turns at P25. These results indicate that *Tmc2* can preserve or prolong survival but cannot preserve function of mature inner HCs in *Tmc1*-deficient mice.

In Tg[P_*Tmc1*_::*Tmc2*]; *Tmc1*^∆/∆^ outer HCs, FM1-43 uptake was observed from P12 to P25. Although current amplitudes were reduced (Fig. 3), the FM1-43 data suggest that transgenic *Tmc2* was sufficient for development and maintenance of functional sensory transduction in *Tmc1*-deficient outer HCs. In contrast, *Tmc2* failed to restore distortion-product otoacoustic emissions (DPOAEs) at P16 in *Tmc1*-deficient mice, indicating that the amplifier function of outer HCs was not restored. The reason for the loss of amplification remains unknown since three aspects known to be required for outer HC function remained intact: (1) expression of the motor protein prestin was not affected; (2) expression of KCNQ4, which mediates *I*_k,n_ in mature outer HCs, appeared normal at P16; and (3) the endocochlear potential was also normal at P16 in Tg[P_*Tmc1*_::*Tmc2*]; *Tmc1*^∆/∆^ cochleae. Finally, outer HC stereocilia bundles remained intact from the apical to basal turns at P16, but started degenerating in the basal turns by P25. We conclude that *Tmc2* was unable to restore the primary function, amplification, or survival of mature cochlear outer HCs in *Tmc1*-deficient mice. Taken together, our results indicate different requirements of inner and outer HCs for TMC1 and TMC2 proteins and different functions of TMC1 and TMC2 in mature cochlear HCs.

Our results from Tg[P_*Tmc1*_::*Tmc2*] mice could have resulted from a loss of expression of *Tmc2* or residual expression of functional *Tmc1* from the Tg[P_*Tmc1*_::*Tmc2*] transgene. However, the results for negative control Tg[*Tmc1*^∆Ex8_9^]; *Tmc1*^∆/∆^ mice showed that residual *Tmc1* was not expressed at functionally significant levels from the Tg[P_*Tmc1*_::*Tmc2*] transgene, which was derived from the same Tg[*Tmc1*^∆Ex8_9^] BAC. The results for the positive control Tg[P_*Tmc1*_::*Tmc1*]; *Tmc1*^∆/∆^ mice showed that the inserted cDNA was expressed at functionally significant levels under the control of the *Tmc1* promoter in the same Tg[*Tmc1*^∆Ex8_9^] BAC used to construct Tg[P_*Tmc1*_::*Tmc2*]. These results indicate that partial restoration of function in Tg[P_*Tmc1*_::*Tmc2*]; *Tmc1*^∆/∆^ mice is unlikely to be due to residual *Tmc1* expression or inadequate expression of *Tmc2* from the BAC transgene. Although we could not directly confirm TMC2 protein expression in cochlear HCs due to difficulty in detecting low levels of protein, the restoration of sensory transduction in Tg[P_*Tmc1*_::*Tmc2*]; *Tmc1*^∆/∆^; *Tmc2*^∆/∆^ HCs indicates that functional TMC2 was expressed from the Tg[P_*Tmc1*_::*Tmc2*] allele.

Why then does TMC2 not fully compensate for loss of TMC1 in mature cochlear HCs? One possible explanation is that TMC1 and TMC2 have different requirements for interacting molecules in mature cochlear HCs. For example, neither TMC1 nor TMC2 binds directly to LHFPL5, but TMC2 and not TMC1 is localized normally to the stereocilia of *Lhfpl5*^∆/∆^ HCs^31^. Thus, the inability of TMC2 to fully substitute for TMC1 may be due to differences in its ability to bind to the correct partners and localize properly at the tips of hair bundle stereocilia.

Another possible explanation for our results is that core properties of the sensory transduction channel, such as single channel conductance and calcium permeability, vary according to the presence of TMC1 or TMC2^17,32^. The properties associated with TMC1, but not TMC2, may be required for the function and survival of mature cochlear HCs. Similarly, another uncharacterized property or function specific to TMC1, but not TMC2, may be required for mature cochlear HC function and survival. One or more of these explanations may account for our current results.

Published studies of the *Beethoven* mouse, which segregates a semi-dominant gain-of-function allele of *Tmc1* (*Tmc1*^*Bth*^), provide additional insight into the differential requirements of inner and outer HCs for TMC proteins^8,13,17^. Heterozygous *Beethoven* mice (*Tmc1*^*Bth*/+^) have delayed onset, progressive HL^13^. On the C3HeB/FeJ strain background, *Tmc1*^*Bth*/+^ inner HC stereocilia bundles degenerate, whereas outer HC stereocilia bundles remained intact at 4 weeks of age^33^. That observation might reflect differing requirements between inner and outer HCs for TMC1/2 for their function and survival. Alternatively, it might reflect differential sensitivity of inner HCs to the gain-of-function effects of the TMC1^Bth^ protein^17^. Furthermore, the DPOAE amplitudes of C3HeB/FeJ-*Tmc1*^*Bth*/+^ mice were significantly higher than those of their hybrid *Tmc1*^*Bth*/+^ offspring of outcrosses to DBA/2J or C57BL/6J mice^33^. We used this background-strain dependent preservation of DPOAE amplitudes and outer HC stereocilia bundles in *Tmc1*^*Bth*/+^ to map modifier loci to chromosomes 2, 5, 11, and 12. The *Tmc1m1* modifier interval on chromosome 2 includes *Tmc2*, raising the possibility that strain-dependent differences in levels of *Tmc2* expression could account for the modifier effect^33^ of alleles at *Tmc1m1* on outer HC function and survival. Finally, the Tg[P_*Tmc1*_::*Tmc1*]; *Tmc1*^∆/∆^ mice in our current study were studied on a C57BL6/J background, suggesting that the loss of amplifier function and failure of cochlear outer HCs to survive might have been less severe or even eliminated on a C3HeB/FeJ background.

In conclusion, transgenic expression of *Tmc2* in mature cochlear HCs partially restored cochlear HC and auditory function in a *Tmc1*-deficient mouse model of DFNB7/B11 deafness. *Tmc1* and *Tmc2* have related but distinct roles in cochlear HCs, and the requirement for *Tmc1* and *Tmc2* differs between inner and outer HCs.

## Methods

### Quantitative RT-PCR analysis

Total RNA was extracted from cochleae or vestibules of C57BL/6J mice or Tg[P_*Tmc1*_::*Tmc2*]; *Tmc1*^∆/∆^ mice using PicoPure RNA Isolation Kit (Life Technologies, Waltham, MA, USA). Total RNA was reverse-transcribed with the SuperScript III First-Strand Synthesis System (Life Technologies). We designed amplification primers specific to *Actb*, *Tmc1*, transgenic and endogenous *Tmc2*, transgenic *Tmc2* or endogenous *Tmc2* cDNA with a ZEN double-quenched probe containing a 5′ FAM fluorophore, a 3′ IBFQ quencher and an internal ZEN quencher (IDT, Coralville, IA, USA; Supplementary Table 1, Fig. 1A). Comparative TaqMan assay was performed using TaqMan Fast Universal PCR Master Mix (Life Technologies) on a ViiA7 Real-Time PCR System (Life Technologies). Each sample was analyzed three times. Relative expression was normalized to the level of actin cytoplasmic 1 (encoded by *Actb*) and calculated using the ΔΔC_t_ method^34^.

### Bacterial artificial chromosome transgenic mice

BAC clone MSMg01-526L10 (181,748 bp, Riken Bioresource Center DNA Bank, Tsukuba, Japan), encoding wild-type MSM/Ms mouse genomic *Tmc1* (170,745 bp)^35^ was used to construct Tg[*Tmc1*^∆Ex8_9^], Tg[P_*Tmc1*_::*Tmc2*] or Tg[P_*Tmc1*_::*Tmc1*] using the Counter-Selection BAC Modification Kit with Red/ET recombination technology (Gene Bridges, Heidelberg, Germany, Fig. 1A). To construct Tg[*Tmc1*^∆Ex8_9^], we deleted 2775 bp including exons 8 and 9 using the same method. This deleted region is identical to that deleted in *Tmc1*^∆/∆^ mice^14^. To construct Tg[P_*Tmc1*_::*Tmc2*] or Tg[P_*Tmc1*_::*Tmc1*], we modified Tg[*Tmc1*^∆Ex8_9^] to replace the translation initiation codon of *Tmc1* with the *Tmc1* (NM_028953.2) or *Tmc2* cDNA (NM_138655.1) conjugated with the SV40 polyadenylation signal, respectively. Mouse cochleae and vestibular organs express two *Tmc1* isoforms, *Tmc1*^Ex1^ and *Tmc1*^Ex2^, which encode transcripts excluding or including exon 2, and utilize a different translation initiation codon in exon 1 or 2, respectively^14^. Since *Tmc1*^Ex1^ can restore MET in *Tmc1*^∆/∆^; *Tmc2*^∆/∆^ cochlear HCs, we replaced the translation initiation codon of *Tmc1*^Ex1^ with cDNA encoding *Tmc1*^Ex1^ or *Tmc2*. Modified BACs were purified and injected into C57BL/6J mouse eggs at the University of Michigan Transgenic Animal Model Core Facility. Founder mice from each BAC construct were backcrossed onto *Tmc1*^∆/∆^ or *Tmc1*^∆/∆^; *Tmc2*^∆/∆^ mice on a congenic C57BL/6J background.

### Genotype analysis

Genomic DNA was extracted from tail clips using the Maxwell 16 System (Promega, Madison, WI, USA). PCR was performed with Taq DNA polymerase (GenScript, Piscataway, NJ, USA) and specific primer sets (Supplementary Table 2) to detect the presence of the transgenes. For *Tmc1*^∆^ genotype analysis, we designed amplification primers specific to the *Tmc1* wild-type allele or the *Tmc1*^∆^ allele (Supplementary Table 3). These primers do not recognize the modified BACs Tg[P_*Tmc1*_::*Tmc2*], Tg[P_*Tmc1*_::*Tmc1*] or Tg[*Tmc1*^∆Ex8_9^].

### Scanning electron microscopy

Samples were prepared from mouse cochleae using the OTOTO method^36^ with some modifications^14^. Essentially, mouse otic capsules were fixed in 2.5% glutaraldehyde buffered with 0.1 M sodium cacodylate buffer (Electron Microscopy Science, Hatfield, PA, USA) containing 2 mM CaCl_2_ and 100 mM sucrose for 1.5 hours at 4 °C. Following washes, samples were post-fixed with 1% osmium tetroxide buffered with 0.1 M sodium cacodylate buffer containing 2 mM CaCl_2_. After cochleae were micro-dissected in 70% ethanol, tissues were gradually hydrated with distilled water. Samples were incubated in a saturated concentration of thiocarbohydrazide for 20 minutes at RT and then washed six times with distilled water. This procedure was repeated twice. The tissues were dehydrated by stepwise incubation in a graded series of increasing concentrations of ethanol, critical point dried, and imaged with an S-4800 Field Emission Scanning Microscope (Hitachi, Tokyo, Japan), at 5-kV acceleration voltage.

### Electrophysiology

Mouse cochlear tissues were harvested at P6-7 and mechanotransduction currents were recorded from outer HCs and inner HCs. The organs of Corti were bathed in standard artificial perilymph containing 137 mM NaCl, 0.7 mM NaH_2_PO_4_, 5.8 mM KCl, 1.3 mM CaCl_2_, 0.9 mM MgCl_2_, 10 mM HEPES, and 5.6 mM D-glucose. Vitamins (1:50) and amino acids (1:100) were added to the solution from concentrates (Invitrogen, Carlsbad, CA, USA), and NaOH was used to adjust the final pH to 7.40 (~310 mOsm/kg). Recording pipettes with resistance of 2–3 MΩ were pulled from R6 capillary glass (King Precision Glass, Claremont, CA, USA). Cs^+^ solution containing 135 mM CsCl, 5 mM HEPES, 5 mM EGTA, 2.5 mM MgCl_2_, 2.5 mM Na_2_-ATP, and 0.1 mM CaCl_2_ was used as intracellular solution, CsOH was used to adjust the final pH to 7.40 (~285 mOsm/kg). Whole-cell, tight-seal, voltage-clamp recordings were performed at −84 mV and 22–24 °C with an Axopatch 200B amplifier (Molecular Devices, San Jose, CA, USA). Hair bundles were deflected with a stiff glass probe with a rounded tip of ~3–5 µm in diameter. The glass probe was mounted on a piezoelectric actuator driven by an LVPZT amplifier (E-500.00, Physik Instrumente, Karlsruhe, Germany). The data were filtered at 10 kHz with a low-pass Bessel filter and digitized at >20 kHz with a 16-bit acquisition board (Digidata 1440 A, Molecular Devices) and pClamp 10 software (Molecular Devices).

### FM1-43 uptake

Mouse otic capsule was harvested and placed in Leibovitz’s L-15 Medium (Life Technologies). FM1-43 FX (Life Technologies), a fixable analog of N-(3-triethylammoniumpropyl)-4-(4-(dibutylamino) styryl) pyridinium dibromide, was applied at 5 µM by local perfusion through oval and round windows for 1 minute at room temperature (RT). After three washes with Leibovitz’s L-15 medium, the tissues were fixed in 4% paraformaldehyde overnight at 4 °C. The organ of Corti was microdissected from the cochlea and counterstained with Alexa Fluor 568 phalloidin (Life Technologies) diluted 1:500. Tissues were mounted with Prolong Gold Antifade without DAPI (Life Technologies) and visualized with an LMS 780 confocal microscope equipped with ZEN 2012 software (Carl Zeiss, Oberkochen, Germany).

### Auditory brainstem response testing

Auditory brainstem response (ABR) thresholds were measured in both ears as described previously^37^. Mice were anesthetized with a mixture of ketamine (56 µg/g) and dexmedetomidine (0.375 µg/g). During the procedure, mice were placed on a heating pad. Eight-, 11.2-, 16-, 22.4- or 32-kHz tone-bursts stimulus of 3-ms duration were delivered at 29.9 Hz. Supra-thresholds stimulus intensities were initially decreased in 10-dB steps, followed by 5-dB steps at lower intensities to determine the thresholds. When no ABR waveform was detectable at the highest intensity level of 90 dB SPL, the threshold was considered to be 95 dB SPL for subsequent analysis.

### Distortion-product otoacoustic emission testing

Distortion-product otoacoustic emissions (DPOAEs) were recorded in both ears as described previously^37^ while the mice were anesthetized for measurement of ABR thresholds. DPOAE levels were obtained in response to two primary tones with frequency ratio f_2_/f_1_ = 1.25 and intensity levels f_1_ = 65 and f_2_ = 55 dB SPL which was varied in one-fifth-octave steps from 4 to 44.8 kHz.

### Endocochlear potential measurement

The endocochlear potential was measured in right ears as described previously^38–40^. Mice were anesthetized with tribromoethanol (350 µg/g) and placed on a heating pad. The endocochlear potential was measured using glass microelectrodes consisting of tips of glass pipettes and silver/chloride electrodes. Glass microelectrodes were prepared using a P-97 Flaming/Brown Micropipette Puller (Sutter Instrument, Novato, CA, USA)^41^. The endocochlear potential was measured in the basal turn of the cochlea by a round-window approach through the basilar membrane. Data were recorded by Digidata 1440 A and AxoScope 10 (Molecular Devices) and analyzed by Clampfit 10 (Molecular Devices).

### Immunohistochemistry

Mouse otic capsules were fixed in 4.0% paraformaldehyde in phosphate-buffered saline (PBS) for 30 minutes at RT. After a 30-minute permeabilization with 0.1% saponin (Sigma-Aldrich, St. Louis, MO, USA), tissues were incubated with blocking solution (2% fetal bovine serum, 5% goat serum and 0.05% saponin in PBS) for 1 hour. Samples were then incubated with primary antibodies in the blocking solution overnight at 4 °C. Following five washes with 0.05% saponin, samples were incubated with secondary antibodies in the blocking solution for 1 hour at RT. After four washes with 0.05% saponin, tissues were washed one time in PBS, and mounted with Prolong Gold Antifade Mountant (Life Technologies). Samples were visualized with an LMS 780 confocal microscope equipped with ZEN 2012 software (Carl Zeiss). The following antibodies were used: goat anti-prestin (1:100; Santa Cruz Biotechnology, Dallas, TX, USA, sc-22692), rabbit anti-myosin-VIIa (1:200; Proteus BioSciences, Ramona, CA, USA, 25–6790), rabbit anti-KCNMA1 (1:100; Sigma-Aldrich, P8357), mouse anti-parvalbumin (1:500, Swant, Bellinzona, Switzerland, 235), rabbit anti-KCNQ4^42,43^ and Alexa Fluor 488- or 568-conjugated secondary antibodies (Life Technologies).

### Statistics

Statistical analyses included paired *t*-test, unpaired Student’s *t*-test and one-way ANOVA. p < 0.05 was considered to be significant.

### Study approval

All animal experiments and procedures adhered to protocols approved by the joint Animal Care and Use Committee of the National Institute of Neurological Disorders and Stroke and the National Institute on Deafness and Other Communication Disorders or by the Animal Care and Use Committee at Boston Children’s Hospital (Protocol #3396).

## Electronic supplementary material

Supplementary Information
