# Supplementary material for: Tmc2 expression partially restores auditory function in a mouse model of DFNB7/B11 deafness caused by loss of Tmc1 function
Source: Sci Rep. 2018 Aug 14;8:12125. doi: 10.1038/s41598-018-29709-8 (PMC6092339; doi:10.1038/s41598-018-29709-8)
Supplement: Supplementary file 1 — Supplementary Information [file 41598_2018_29709_MOESM1_ESM.pdf]

Supplementary Information

Tmc2 expression partially rescues auditory function in a mouse model of DFNB7/B11 deafness caused by loss of *Tmc1* function

Hiroshi Nakanishi[1], Kiyoto Kurima[1], Bifeng Pan[2], Philine Wangemann[3], Tracy S. Fitzgerald[4], Gwenaëlle S. Géléoc[2], Jeffrey Holt[2], Andrew J. Griffith[1*]

1. Otolaryngology Branch, National Institute on Deafness and Other Communication Disorders (NIDCD), NIH, Bethesda, Maryland 20892, USA
2. Department of Otolaryngology and Neurology, F. M. Kirby Neurobiology Center, Boston Children's Hospital, Harvard Medical School, Boston, Massachusetts 02115, USA
3. Anatomy and Physiology Department, Kansas State University, Manhattan, Kansas 66506, USA
4. Mouse Auditory Testing Core Facility, NIDCD, NIH, Bethesda, Maryland 20892, USA

Correspondence should be addressed to A.J.G. (email: griffita@nidcd.nih.gov)

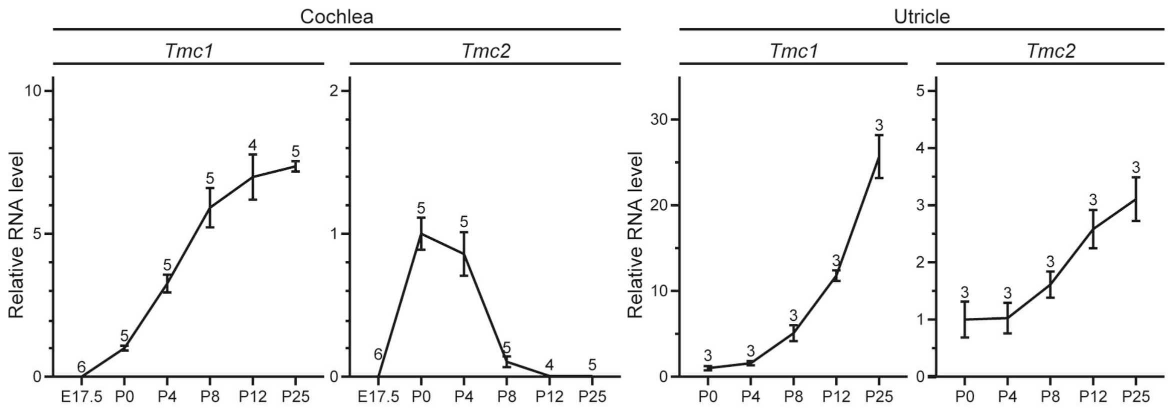

Supplementary Figure 1. *Tmc1* and *Tmc2* mRNA levels (mean ± SD) of wild-type mouse cochleae and vestibules. *Tmc1* expression was persistent in mature cochlear and vestibular hair cells. Whereas, *Tmc2* expression was transient in early postnatal cochlear hair cells but persisted in vestibular hair cells. The number above each data point and bar indicates the number of mice examined. The mRNA level was first normalized to the *Actb* level and then to its own value measured at P0.

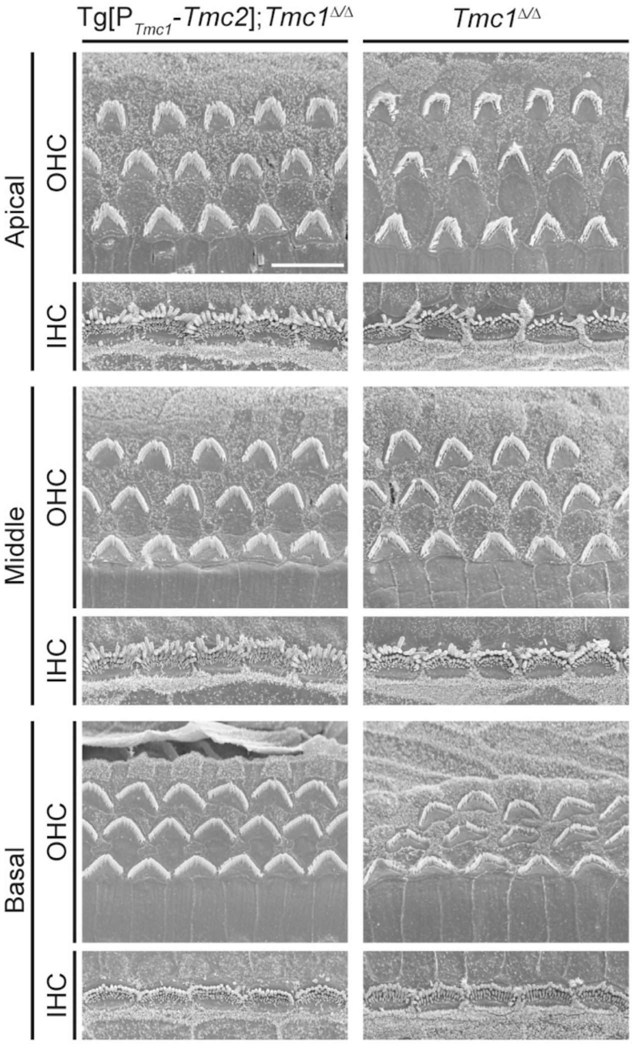

Supplementary Figure 2. Hair cell morphology of transgenic mice examined by scanning electron microscopy at P16. Inner and outer hair cell stereocilia bundles of Tg[P$_{Tmc1}$::*Tmc2*]*Tmc1*$^{\Delta/\Delta}$ (n=4 mice) cochleae remained intact from apical to basal turns, while outer hair cell stereocilia bundles of *Tmc1*$^{\Delta/\Delta}$ (n=3 mice) cochleae are starting to degenerate from middle to basal turns (Scale bar, 10 μm).

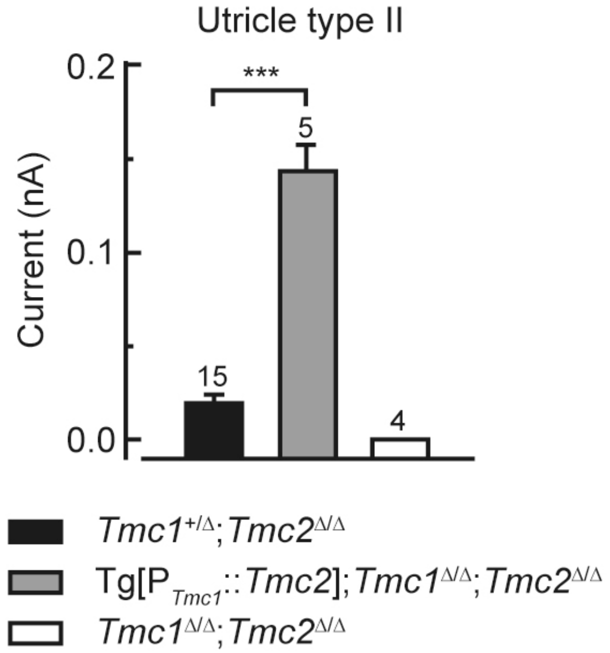

Supplementary Figure 3. Whole cell sensory transduction currents of Tg[P*Tmc1*::*Tmc2*];*Tmc1*$^{\Delta/\Delta}$;*Tmc2*$^{\Delta/\Delta}$ vestibular HCs at P4-8. The sensory transduction currents of utricle type II hair cells in Tg[P*Tmc1*::*Tmc2*];*Tmc1*$^{\Delta/\Delta}$;*Tmc2*$^{\Delta/\Delta}$ are significantly larger than those of *Tmc1*$^{+/\Delta}$;*Tmc2*$^{\Delta/\Delta}$ mice (paired *t*-test, $P < 0.001$). The number above each column and bar indicates the number of HCs examined.

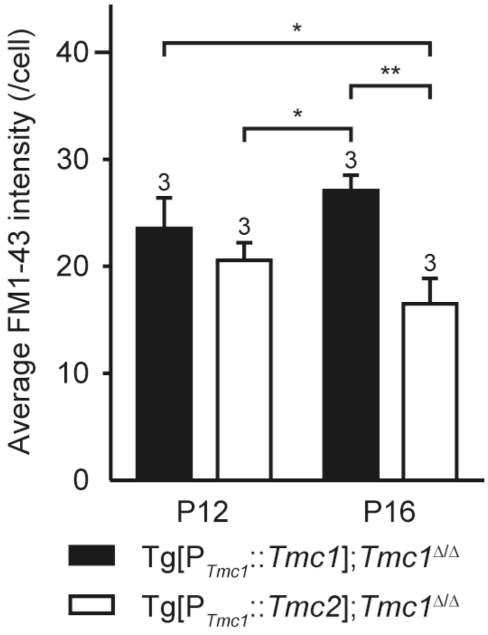

Supplementary Figure 4. Average FM1-43 intensity in inner HCs of Tg[P$_{Tmc1}$::*Tmc2*];*Tmc1*$^{\Delta/\Delta}$ cochleae. The average intensity in inner HCs of Tg[P$_{Tmc1}$::*Tmc2*];*Tmc1*$^{\Delta/\Delta}$ cochleae is significantly smaller than that of Tg[P$_{Tmc1}$::*Tmc1*];*Tmc1*$^{\Delta/\Delta}$ cochleae at P16 (one-way ANOVA, $P < 0.005$). Quantitation of FM1-43 intensity was performed using ImageJ (http://rsbweb.nih.gov/ij/). The number above each column and bar indicates the number of mice examined.

Supplementary Table 1. Nucleotide primers for quantitative RT-PCR

Target gene	Primer/Probe sequence (5' to 3')	Location of primer/probe
Actb	F: ACCTTCTACAATGAGCTGCG	Exon 3
	R: CTGGATGGCTACGTACATGG	Exon 4
	P: /FAM/TCTGGGTCA/ZEN/TCTTTTCACGGTTGGC/IBFQ/	Exons 3-4
Tmc1	F: GGTGCTGGGACTTAGAATATGG	Exon 15
	R: TGCCTTGGTTGAAGATCAGAG	Exon 16
	P: /FAM/AGGACGTTG/ZEN/CCACTGATGTCGAAT/IBFQ/	Exon 16
Tmc2[A]	F: CATCCTGCTAATGTTTCTGGC	Exons 17-18
	R: ACTATGGCTTTTCCCTTGATGG	Exon 19
	P: /FAM/TGGTTCTTC/ZEN/TCAACTTCACGGAGCG/IBFQ	Exon 18-19
Exogenous *Tmc2*	F: GGTCACCTTCTGGAAAGAGAA	Exon 20
	R: TTTTTAAAGCAAGTAAAACCTCTACAA	3' UTR of inserted *Tmc2*
	P: /FAM/TGGCTGATT/ZEN/ATGATCTAGAGTCGCGGC/IBFQ/	3' UTR of inserted *Tmc2*
Endogenous *Tmc2*	F: GTCTCAGACTTACACAGGCAG	Exon 20
	R: ACAGTAGAGATTCAAGCCAAGG	3' UTR of *Tmc2*
	P: FAM/CTGATTTTC/ZEN/TGGCATTCATGGGTGTCC/IBFQ	3' UTR of *Tmc2*

Abbreviations: F, forward primer; R, reverse primer; P, probe.
[A]The set of primers amplify exogenous and endogenous *Tmc2*.

Supplementary Table 2. Nucleotide primers for genotyping of transgenic mice

	Primer sequence (5' to 3')	Target transgene
Tmc1 BAC$^{\Delta Ex8-9}$	F: GCATCCTAGGAAATACCGAAATAC	Tg (*Tmc1*-*Tmc2*), Tg (*Tmc1*-*Tmc1*),
	R: GGTTTGCTAAGGTGACAAGTACAA	Tg (*Tmc1*$^{\Delta Ex8-9}$)
BAC SP6	F: GCACAACACATGTTTATTCACTCA	Tg (*Tmc1*-*Tmc2*), Tg (*Tmc1*-*Tmc1*),
	R: CCGTCGACATTTAGGTGACACTAT	Tg (*Tmc1*$^{\Delta Ex8-9}$)
BAC T7	F: GGCCGCTAATACGACTCACTAT	Tg (*Tmc1*-*Tmc2*), Tg (*Tmc1*-*Tmc1*),
	R: AGTAGAGTCCAGGCATGCTAAAAT	Tg (*Tmc1*$^{\Delta Ex8-9}$)
Inserted *Tmc1* cDNA	F: ACAAGAAAATGGCAGCGGCTCGAG	Tg (*Tmc1*-*Tmc1*)
	R: TTTGGCATGTATATCTAAACAGCATAG	
Inserted *Tmc2* cDNA	F: CAGACCCTGGACAAGAAAGCGCAG	Tg (*Tmc1*-*Tmc2*)
	R: TTTGGCATGTATATCTAAACAGCATAG	

Abbreviations: F, forward primer; R, reverse primer.

Supplementary Table 3. Nucleotide primers for genotyping of *Tmc1* knockout mice

Primer set	Primer sequence (5' to 3')
Tmc1 wild-type allele	F: GCATCCTAGGAAATACCGAAATAC
	R: GAAGAATTTTGAAAGCAAATCTGA
Tmc1 knockout allele	F: GCATCCTAGGAAATACCGAAATAC
	R: AAACATACTTTCGGTTCCTCTTC

Abbreviations: F, forward primer; R, reverse primer.
